# Low Serum Creatine Kinase Levels in Breast Cancer Patients: A Case-Control Study

**DOI:** 10.1371/journal.pone.0062112

**Published:** 2013-04-15

**Authors:** Hong Pan, Kai Xia, Wenbin Zhou, Jinqiu Xue, Xiuqing Liang, Lin Cheng, Naping Wu, Mengdi Liang, Dan Wu, Lijun Ling, Qiang Ding, Lin Chen, Xiaoming Zha, Xiaoan Liu, Shui Wang

**Affiliations:** Department of Breast Surgery, The First Affiliated Hospital with Nanjing Medical University, Nanjing, China; Okayama University, Japan

## Abstract

**Background:**

Previous studies provide an ambiguous picture of creatine kinase (CK) expression and activities in malignancy. The aim of this study was to investigate the role of serum CK level in breast cancer patients.

**Patients and Methods:**

823 female patients diagnosed with breast cancer were consecutively recruited as cases, and 823 age-match patients with benign breast disease were selected as controls. Serum CK was analyzed by commercially available standardized methods.

**Results:**

Serum CK level was significantly associated with breast cancer (*P* = 0.005) and subtypes of breast cancer, including breast cancer with diameter>2 cm (*P* = 0.031) and stage IIIbreast cancer (*P* = 0.025). The mean serum CK level in patients with>2 cm tumor was significantly lower than that in≤2 cm (*P* = 0.0475), and the mean serum CK level of stage III breast cancer patients was significantly lower than that of stage I and II breast cancer patients (*P* = 0.0246). Furthermore, a significant difference (*P* = 0.004) was observed between serum CK level and ERBB2+breast cancer not other molecular subtypes.

**Conclusions:**

Serum CK levels in cases was significantly lower compared with controls. Notably, our results indicated for the first time that there was a negative correlation between serum CK levels and breast cancer stage. Serum CK level, which may reflect the status of host immunity, may be an important factor in determining breast cancer development and progression.

## Introduction

Breast cancer is a common malignancy and the second leading cause of cancer death among women [1]. In addition, the incidence of breast cancer continues to increase [2], [3]. Previous studies have estimated that approximately 50% of breast cancer can be attributed to well-known risk factors, such as age, family history, endocrine factors, and host factors [4], [5]. Besides, it is suggested that the immune system is involved in cancer development and progression.

A recent review implicates that immune response is a unifying and non-site specific determinant of cancer risk [6]. Epidemiological studies indicate that metastasis might be initiated already 5–7 years before diagnosis of the primary breast cancer, yet only a subset of patients develop clinically evident relapse [7]. It is speculated that the host immune response plays an important role in suppressing metastasis of breast cancer.

Creatine kinase (CK), an enzyme, is expressed by various cells and tissues [8]. In vertebrates, two cytosolic and two mitochondrial CK isoenzymes have been identified. The cytosolic CK isoforms can exist as homodimers (CK-MM and CK-BB) or a heterodimer (CK-MB). CK plays a pivotal role in energy transduction in tissues with high and fluctuating energy demands, particularly in cardiac and skeletal muscle [9], [10], [11]. Importantly, previous studies have demonstrated that CK may play an important role in immune response, including adaptive immune response and innate immune response. A previous study has demonstrated that CK-BB is an important regulator of T cell development and activation via TCR signaling [12]. What's more, CK is one of the most important biomarkers which may reflect the status of innate immunity. Previous studies indicate that amphioxus fluid CK played the immune role as an acute phase protein, and humoral fluid CK downregulated to give feedback for regulation of innate immunoprotection [13], [14]. Therefore, we speculate that CK may take part in malignancy occurrence and progression through acting in immune response.

However, previous studies provide a somewhat ambiguous picture of CK expression and activity in malignant cells and tumor-bearing animals. Some studies report the upregulation of some isoforms of CK in malignant cells [15], [16], [17]. By contrast, some other studies suggest a decrease in the activity of CK and some of its isoforms in different malignancies [18], [19], [20], [21], [22]. Moreover, results of previous clinical studies are also inconsistent [23], [24]. To date, the field of CK in relation to malignancy remains under intense investigation.

To the best of our knowledge, few large epidemiological studies have addressed the association between serum CK levels and breast cancer or subtypes of breast cancer until now. The present case-control study was conducted to determine the value of serum CK in breast cancer based on relatively larger samples. The association between serum CK levels and subgroups of breast cancer (different tumor stage, tumor size, lymph node involvement and molecular subtype) was also investigated.

## Materials and Methods

### Study population

The present case-control study was carried out in the First Affiliated Hospital with Nanjing Medical University. It was approved by the ethics committee of our hospital. All patients provided written informed consent for their clinical information to be reviewed by us. This study was in compliance with the Helsinki Declaration.

The patients were newly diagnosed with histologically confirmed breast cancer from January 2006 to June 2011. Histological sections of all cases were reviewed by two pathologists independently. All cases were consecutively recruited without restriction of age or histological type. And all the subjects in the present study are ethnic Han Chinese coming from different families and have no blood relationship. Patients who were diagnosed with histologically confirmed specific invasive ductal carcinoma were excluded from this study due to small sample size. What's more, patients with metastasis, or prior history of other malignancies, or skeletal and cardiac muscle disease or brain disease, or liver and renal dysfunction were excluded from this study. In addition, none of the participants was receiving any drug that could influence circulating CK level. A total of 823 female patients (mean age 50.1 years, range 23–83 years) with incident breast cancer were included as cases. For each case, one age-matched control(±5 years) was chosen from patients (mean age 49.9 years, range 23 to 83 years) with histologically confirmed benign breast disease in the same period.

### Data collection

Potential risk factors and clinical information were obtained by trained interviewers. The following potential risk factors were collected if available: age at diagnose, age at menarche, previous childbearing, menopausal status, diabetes mellitus, and hypertension. Clinical information was also obtained, including pathology (ductal carcinoma *in situ* (DCIS) or invasive breast cancer), breast cancer stage (0 to III), tumor size (≤2 cm or >2 cm), lymph node involvement (negative or positive), histological grade (I to III), hormone receptor status (negative or positive), and molecular subtype (HR+/ERBB2-, triple negative, or ERBB2+).

For invasive cancers, Her2 status was checked by immunohistochemistry or FISH. If the result of immunohistochemistry was +++ or Her2 was amplified confirmed by FISH, Her2 was difined as overexpression. Otherwise, Her2 was defined as non-overexpression. ER and/or PR positive were considered as hormone receptor positive, while both ER and PR negative were considered as hormone receptor negative. HR+/ERBB2- breast cancer was defined as hormone receptor positive and Her2 negative. Triple-negative breast cancer was defined as ER, PR and Her2 were all negative. Otherwise, ERBB2+ breast cancer was defined as Her2 overexpression regardless of hormone receptor status in the present study.

### Laboratory studies

Antecubital venous blood samples were collected from all subjects after fasting overnight. Blood samples were obtained from breast cancer patients before operation, chemotherapy, or other therapies. CK was analyzed by commercially available standardized methods.

### Statistical analysis

Numerical data were reported as means ± standard deviation (SD). Multivariate logistic regression was used to estimate odds ratio (OR) and 95% confidence interval (CI) for the association between serum CK levels and breast cancer risk. The analyses of ORs was adjusted for age at diagnose, age at menarche, childbearing, menopause status, diabetes mellitus, and hypertension. One-way ANOVA was used to identify differences of serum CK levels among different subgroups of breast cancer. All statistical analyses were performed by using statistics software (State version 11.0, State), and all *P*-values were two-tailed with 5% significance levels.

## Results

The clinical characteristics of all subjects are summarized in Table 1. It presents the distribution of breast cancer cases and benign breast disease controls according to age, menarche age, childbearing, menopausal status, and other selected covariates. Of these 823 breast cancer patients, 24 patients were diagnosed with DCIS, and 799 with invasive breast cancer according to the NCCN guideline. Cases and controls were well matched on age (*P* = 0.921). In addition, no significant differences were observed between cases and controls with regards to menarche age and diabetes mellitus (*P* = 0.2189 and *P* = 0.954, respectively). However, compared with benign breast disease controls, more postmenopausal subjects (*P* = 0.004), fewer hypertension subjects (*P* = 0.036) were reported in breast cancer cases. Furthermore, significant difference of childbearing status was observed between the two groups (*P* = 0.0471).

**Table 1:** Clinical characteristics of the study population.

Variables	Cases (n = 823)		Controls (n = 823)		*P* *
	No.	%	No.	%	
Ages (mean, range)	50.1, 23∼83		50.0, 23∼83		
≤50	453	55.0	455	55.3	0.921
>50	370	45.0	368	44.7	
Menarche ages (years)					
≤13	126	15.3	133	16.2	0.2189
14∼16	361	43.9	442	53.7	
≥17	117	14.2	94	11.4	
NA	219	26.6	154	18.7	
Childbearing					
0	22	2.7	34	4.1	**0.0471**
1	442	53.7	511	62.1	
2	158	19.2	138	16.8	
≥3	57	6.9	63	7.7	
NA	144	17.5	77	9.4	
Status menopausal					
Premenopause	388	47.1	480	58.3	**0.004**
Postmenopause	330	40.1	302	36.7	
NA	105	12.8	41	5.0	
Diabetes mellitus					
No	728	88.5	769	93.4	0.954
Yes	43	5.2	46	5.6	
NA	52	6.3	8	1.0	
Hypertension					
No	664	80.7	673	81.8	**0.036**
Yes	107	13.0	145	17.6	
NA	52	6.4	5	0.6	

* Nonparametric rank test for menarche age and childbearing distribution between cases and controls; Two-sided x^2^ test for other variables distribution between cases and controls; NA, not available.

### The mean serum CK levels in subtypes of breast cancer

The mean serum CK levels in breast cancer cases stratified according to several clinical variables as presented in Table 2. It is observed that there were no significant differences in serum CK levels among breast cancer patients with different pathology (*P* = 0.5687), grade (*P* = 0.5260), hormone receptor status (*P* = 0.3557), and molecular subtype (*P* = 0.1992). Furthermore, a borderline significant difference in serum CK levels was observed between breast cancer patients with or without lymph node involvement (*P* = 0.0687). Interestingly, we found that the mean serum CK level in patients with>2 cm tumor was significantly lower than that in patients with≤2 cm tumors (72.95±34.20 U/L and 78.40±38.23 U/L, respectively, *P* = 0.0475). Moreover, patients with stage III breast cancer also showed a significantly lower serum CK levels than patients with stage I and II breast cancer (69.95±32.52 U/L and 77.04±36.94 U/L, respectively, *P* = 0.0246).

**Table 2:** Means (SD) of serum CK in the study population.

Variables	Cases (n)	Mean (SD)	*P* ^#^
Pathology			
DCIS	24	79.57(49.63)	0.5687
Invasive cancer	799	75.24(35.97)	
Stage			
I-II	520	77.04(36.94)	**0.0246**
III	175	69.95(32.52)	
Tumor size*			
≤2 cm	309	78.40 (38.23)	**0.0475**
>2 cm	399	72.95 (34.20)	
Lymph node involvement*			
Negative	436	75.87 (35.61)	0.0687
Positive	345	71.18 (35.31)	
Grade*			
I-II	361	75.25 (37.48)	0.5260
III	238	73.33 (33.67)	
Hormone receptor status*			
Negative	207	72.42 (35.48)	0.3557
Positive	500	75.18 (36.08)	
Molecular subtype*			
HR+/ERBB2-	399	76.19 (36.80)	0.1992
Triple negative	132	74.07 (35.35)	
ERBB2+	165	70.26 (32.62)	

* DCIS not included for analysis; ^#^One-way ANOVA used for analysis.

### Multivariate analysis of breast cancer risk

Multivariate logistic regression analysis was applied to evaluate the relationship between selected variables and the risk of breast cancer (DCIS and invasive breast cancer included). ORs and 95% CIs of clinical variables for breast cancer are shown in Table 3. There was a significant association between breast cancer risk and decreased serum CK levels (OR = 0.9955, 95% CI = 0.9924–0.9986, *P* = 0.005). Postmenopausal patients showed a significantly higher breast cancer risk than premenopausal patients (OR = 3.18, 95% CI = 2.04–4.97, *P*<0.001). However, no significant association was observed between breast cancer risk and other clinical variables (all *P*>0.05), including age at diagnose, age at menarche, previous childbearing, diabetes mellitus and hypertension.

**Table 3:** Multivariate logistic analysis of breast cancer risk factors.

Variables	OR	95% CI	*P*
CK	0.9955	0.9924–0.9986	**0.005**
Age at diagnose			
≤30	Reference		
31–40	1.11	0.41–3.01	0.839
41–50	0.88	0.33–2.36	0.799
51–60	0.50	0.17–1.46	0.205
61–70	0.39	0.12–1.23	0.108
71–80	0.41	0.12–1.45	0.167
≥81	0.49	0.05–4.76	0.534
Age at menarche			
≤13	Reference		
14–16	0.83	0.61–1.14	0.247
≥17	1.22	0.81–1.84	0.335
Childbearing			
0	Reference		
1	1.26	0.68–2.34	0.466
2	1.56	0.79–3.05	0.795
≥3	1.16	0.54–2.48	0.707
Menopause status			
Premenopause	Reference		
postmenopause	3.18	2.04–4.97	**<0.001**
Diabetes mellitus			
No	Reference		
Yes	1.14	0.66–1.95	0.645
Hypertension			
No	Reference		
Yes	0.74	0.51–1.08	0.117

### Risk of subtype-specific breast cancer in relation to serum CK

Furthermore, we evaluated the association between serum CK levels and breast cancer risk stratified by breast cancer stage, tumor size, lymph node involvement, grade, hormone receptor status, and molecular subtype. As shown in Table 4, significant association between serum CK levels and breast cancer risk was observed regardless of lymph node involvement, grade and hormone receptor status (all *P*<0.05). In multivariate logistic analysis, there was a significant association between serum CK levels and breast cancer with diameter>2 cm (OR = 0.9955, 95% CI = 0.9915–0.9996, *P* = 0.031), but not breast cancer with diameter≤2 cm (OR = 0.9967, 95% CI = 0.9926–1.0007, *P* = 0.105). Furthermore, a significant association was also observed between serum CK levels and stage III breast cancer (OR = 0.9928, 95% CI = 0.9865–0.9991, *P* = 0.025), but not stage 0 (OR = 1.00, 95% CI = 0.9962–1.0113, *P* = 0.331), stage I(OR = 0.9948, 95% CI = 0.9896–1.0000, *P* = 0.055), and stage II (OR = 0.9977, 95% CI = 0.9940–1.0015, *P* = 0.240) breast cancer. For molecular subtype, the significant association between serum CK levels and ERBB2+breast cancer was observed (OR = 0.9907, 95% CI = 0.9843–0.9971, *P* = 0.004), and no significant association was observed between serum CK levels and the other two subtypes of breast cancer (both *P* = 0.123).

**Table 4:** Risk of subtype-specific breast cancer in relation to serum CK.

Variables	OR	95% CI	*P* ^#^
Stage			
0	1.00	0.9962–1.0113	0.331
I	0.9948	0.9896–1.0000	0.055
II	0.9977	0.9940–1.0015	0.240
III	0.9928	0.9865–0.9991	**0.025**
Tumor size*			
≤2 cm	0.9967	0.9926–1.0007	0.105
>2 cm	0.9955	0.9915–0.9996	**0.031**
Lymph node involvement*			
Negative	0.9957	0.9919–0.9996	**0.030**
Positive	0.9937	0.9892–0.9983	**0.007**
Grade*			
I-II	0.9959	0.9920–0.9998	**0.042**
III	0.9932	0.9873–0.9990	**0.023**
Hormone receptor status*			
Negative	0.9924	0.9870–0.9979	**0.006**
Positive	0.9963	0.9928–0.9999	**0.045**
Molecular subtype*			
HR+/ERBB2-	0.9971	0.9934–1.0007	0.123
Triple negative	0.9951	0.9889–1.0013	0.123
ERBB2+	0.9907	0.9843–0.9971	**0.004**

* DCIS not included for analysis; ^#^Adjusted for age at diagnose, age at menarche, childbearing, menopause status, diabetes mellitus, and hypertension.

## Discussion

The present study is the largest available study on the association of serum CK levels and breast cancer. Our results revealed that serum CK levels in breast cancer patients were significantly lower compared with patients with benign breast disease. Significant association between serum CK levels and breast cancer was observed regardless of lymph node involvement, grade and hormone receptor status. Notably, our results indicated for the first time that there was a negative correlation between serum CK levels and tumor size. The same negative correlation was also observed between serum CK levels and breast cancer stage. In subgroup multivariate analysis, serum CK levels were significantly associated with breast cancer with larger tumor size(>2 cm) and advanced stage (stage III). Furthermore, serum CK levels were significantly associated with ERBB2+ breast cancer not HR+/ERBB2- or triple negative breast cancer.

CK plays a key role in the energy homeostasis of excitable cells by the connecting the sites of ATP generation and consumption and buffering the intracellular ATP/ADP ration [25]. It participates in many vital physiological processes [12], [26], [27], [28], [29], [30]. Interestingly, it is worth noting that several studies demonstrated dysregulation of CK in different malignancies. Brett Delahunt and colleges reported the clinical usefulness of CK and the isoenzyme CK-MB as tumor markers for Wilms tumor in two cases. They found that serum CK and CK-MB levels were significantly elevated in both cases [23]. On the contrary, another study suggested that CK activity gradually decreased progressively in the muscles with the progression of malignancy and even non-detectable in the final stage of dedifferentiation. Low levels of CK may represent a general trend in cancer favoring pathways towards tumorigenesis [24]. It will be interesting to determine whether the downregulation of CK could be observed in other cancers growing in non-muscle tissue [31].

Previous studies provide an ambiguous picture of CK expression and activities in breast cancer [32], [33], [34]. However, none of previous studies is a large epidemiological study investigating the association between serum CK levels and breast cancer. In the present study, we demonstrated for the first time that serum CK levels decreased in breast cancer patients in a case-control study with relative large sample size. However, the role of serum CK in breast cancer remains unclear and the mechanisms responsible for lower serum CK levels needs to be elucidated.

We hypothesize that low serum CK levels in breast cancer cases may attribute to the host immune response. It is known to all that the immune system protects the host from the infection or disease by innate and adaptive responses [35]. Furthermore, immune response is an important factor in determining tumor development, progression and metastasis, contributing to tumor escape and failure of therapies [36], [37]. Importantly, previous studies suggested CK was considered to take part in immune response. As reported, CK-BB was demonstrated to be an important regulator of T cell development and activation [12]. The intracellular ATP concentration and the ATP regeneration capacity of T cells were considered to have strong impact on TCR signal strength [38], [39]. Moreover, it has been reported that CK helps keep the ATP pool constant [40], [41], [42]. Therefore, we infer that CK takes an important part in immune response affecting breast cancer development and progression, and decreased serum CK levels may reflect the status of immunity of the host.

Interestingly, there was a negative correlation between serum CK levels and tumor size or breast cancer stage. So it is speculated that the lower the CK levels, the poorer the host immunity to malignancy was, contributing to tumor progression. As reported, CK is an important regulator of T cell development and activation which is an important factor in immune response. It is reported that ectopic expression of CK-BB led to increase ATP level and enhance phosphorylation of the TCR signaling proteins. CK-BB was demonstrated to enhance the magnitude of tyrosine phosphorylation of Lck and Zap70, and to increase the phosphorylation of p38, JNK and Erk1/2 in the presence of TCR stimulation. In addition, TCR-induced Ca^2+^ mobilization was also enhanced. And the activation, proliferation and cytokine secretion of T cells were also enhanced by the expression of CK-BB. Thus, CK-BB plays an important role in regulating thymocyte development and T cell activation via modulating TCR signal strength [12]. T lymphocytes infiltrate extensively to higher grade DCIS and invasive carcinomas [43]. Intrathymic T cell development is critical for the establishment of a properly functioning immune response. In rapidly proliferating tumors, the presence of T lymphocytes at tumor sites is a good prognostic indicator when compared with nonimmunogenic tumors, and correlates with auxillary lymph node negativity, a smaller tumor diameter and a lower histological grade [44]. Therefore, low CK levels may reflect the poor immune response status, thus cause breast cancer development and progression, like advanced stage and large tumor size. However, in our present study, a borderline significant difference of serum CK levels was observed between patients with lymph node positive and negative due to small sample size.

In addition, it is observed for the first time that serum CK levels were significantly associated with ERBB2+ breast cancer, but not other molecular subtypes. Since 1994, several studies have shown the presence of immune response against Her2 in patients with ERBB2+ breast cancer [45]. The immune response can be associated with slower tumor development at early stage of the disease [46]. We may assume that immune response may play a specific role in ERBB2+ breast cancer, relating to serum CK. However, the exact underlying mechanisms are not clear, and should be investigated in the future.

There are some limitations in our study. First, as a retrospective case-control study, we can't rule out research bias. Second, although our present study is the largest available to date on the association of serum CK levels and breast cancer, the sample size is still relatively small. Third, the causal relationship between serum CK levels and breast cancer is not exact clear. We hypothesized that low serum CK levels may lead to breast cancer development and progression, but future prospective study with larger sample size should be carried to draw definitive conclusions.

In conclusion, the present study is the largest one to investigate the association between serum CK levels and breast cancer. Our results revealed that the serum CK levels in breast cancer patients, especially ERBB2+ breast cancer, were significantly lower compared with patients with benign breast disease. And there was a negative correlation between CK levels and breast cancer stage or tumor size. Low serum CK level may be a marker of advanced breast cancer. The present study seemed to support a protective effect of serum CK for breast cancer. Furthermore, serum CK level is supposed to be used as an important factor in determining breast cancer development and progression. However, future experimental and prospective studies are warranted to confirm the results.
